# Commentary: Patanjali and neuroscientific research on meditation

**DOI:** 10.3389/fpsyg.2018.00248

**Published:** 2018-02-27

**Authors:** J. Shashi Kiran Reddy, Sisir Roy

**Affiliations:** ^1^Research Scholar, Bangalore, India; ^2^Consciousness Studies Programme, National Institute of Advanced Studies, Indian Institute of Science Campus, Bangalore, India

**Keywords:** patanjali, meditation research, mental noise, Buddhahood, meditative states of consciousness

In Bærentsen (2015), various issues have been raised mainly concerning the contemporary view of the practice of meditation with respect to the actual philosophical perspective. We endorse the idea of referring to ancient traditional sources on meditation—as a practice and thus are in agreement with the many of the points discussed in Bærentsen (2015). In contrast to modern scientific experiments on meditation, the practice of meditation is not conceived either for the enhancement of cognitive functions or for the purpose of well-being (Rao, 2011; Awasthi, 2013; Nash and Newberg, 2013; Schmidt, 2014; Tomasino et al., 2014; Bærentsen, 2015), though it appears to show the promising influence on these aspects (Ospina et al., 2007; Braboszcz et al., 2010; Schmidt and Walach, 2014; Tang et al., 2015; Tomasino and Fabbro, 2015). It is utilized mainly as a tool to realize one's true nature by attaining Buddhahood or Samadhi or any similar experiential state as quoted in different spiritual traditions (which we refer to here as a natural meditative state for a valid reason: Woods, 1927/2003; Rao, 2011; Schmidt, 2014; Schmidt and Walach, 2014; Tomasino et al., 2014). Such state can be looked upon as a subjective experience devoid of mental fluctuations or noises; which is close to the experience of thoughtless state (as indicated in the traditional scriptures; Woods, 1927/2003; Rukmani, 2001).

As indicated by Patanjali, there are types of mental fluctuations (or mental noises) that one has to overcome to reach a stable meditative state (which are discussed in detail elsewhere; Woods, 1927/2003; Rukmani, 2001; Rao, 2011; Awasthi, 2013; Bærentsen, 2015). These fluctuations/noises are known to hinder one's potential to realize a natural meditative state that is common to everyone. Besides, he goes along to say that one's natural state is not something that has to be attained through a technique or a process, the ultimate meditative state that every meditator aspires for is already with us and we just have to clear out the noise that blocks the way to realization. On careful observation, we see that each type of noise is associated with different physical mechanisms and cognitive functions (Bærentsen, 2015); hence each requires a different technique to deal with and overcome. Consequently, we have numerous techniques and types of meditations across traditions.

The various techniques and modalities of meditation suggested in the respective traditions involve clearing out the fluctuations caused by numerous faculties. A deeper insight into various types and stages associated with each of these techniques reveal that each different type of meditation involves a process to reduce any of the noise components and gradually takes through different stages of evolvement to reach the same and ultimate experiential state (Woods, 1927/2003; Rukmani, 2001; Braboszcz et al., 2010; Rao, 2011; Schmidt, 2014). Such an observation is needed and would play a crucial role in sorting out various confusions caused by definitional and taxonomical issues (Rao, 2011; Awasthi, 2013; Nash and Newberg, 2013). Regardless, when we attempt to look at each type of meditation as a separate practice by itself, as done in most meditation studies (though there have been other attempts; Fingelkurts et al., 2016a,b), it leads to misinterpretations and incomplete views. This is one obvious reason why research studies on different types of meditation report distinct results and observations.

The dynamics of how an individual responds and benefits from meditation practice depends on his current evolutionary state which is driven by both the inherent and acquired personality traits and external influences etc. (Fingelkurts et al., 2015, 2016b; Tang et al., 2015, 2016). Thus, this indicates that each person will have varying noise levels that are unique to his own personality. If that is the case, then how can a mediator's level of expertise be based and studied in comparison to a novice or other individual? The better choice would be to study a subject during various states say from waking state to deep sleep state and then draw a conclusion on his state of meditative experience (Cvetkovic and Cosic, 2011; Travis, 2011; Dissanayaka et al., 2015). To overcome this, we suggest a new baseline, which is universally present in every individual derived from the above-discussed perspective. Here, we emphasize that a “universal baseline” may exist in the very assumption that Buddhahood is present in every individual and people meditate to realize this state in their experience. So a universal baseline can be defined only in terms of Buddhahood or natural meditative state. Meditation then can be treated as a plumbing process to reduce and clear the fluctuations/noises developed over the course of one's evolution. A challenging issue for future studies is to figure out how to define and capture such state for an operational purpose. We can call this a noise free model of meditation and here, noise is an unwanted variation in terms of the fluctuations of the mind.

From this viewpoint, all the data emerging from different studies on meditation are essentially indicating various levels and extent of noise. That is the reason why each type of meditation involves different levels of coherence and neural patterns (Travis et al., 2010; Tomasino et al., 2014; Fingelkurts et al., 2016a; Braboszcz et al., 2017). From the contemporary view of meditation in science, one can never reach an agreement with the real purpose of different types of meditations across traditions and cultures. All studies would simply report more and more data, but won't serve our actual purpose of understanding the phenomenon of meditation in its totality. Various interpretations and comparative studies on meditation are then like comparing one type of noise to the other. One has to see how future studies on meditation would embed such ideas that are crucial to giving us a better understanding of our true purpose of life.

## Author contributions

JR came up with an idea to write comments and extend the original article. Both JR and SR analyzed and wrote the article.

### Conflict of interest statement

The authors declare that the research was conducted in the absence of any commercial or financial relationships that could be construed as a potential conflict of interest.
